# Proximal Ureteral Atresia in a Caribbean Neonate With Multicystic Dysplastic Kidney

**DOI:** 10.7759/cureus.89113

**Published:** 2025-07-31

**Authors:** Paige V Ali, Fiaz Ali, Lakhan Roop

**Affiliations:** 1 Paediatric Surgery, San Fernando Teaching Hospital, San Fernando, TTO

**Keywords:** large intra‑abdominal mass, multicystic dysplastic kidney, open nephrectomy, paediatric surgery, ureter anomaly

## Abstract

Proximal ureteral atresia is an exceedingly rare presentation of congenital or neonatal hydronephrosis. We report the case of a full-term, female neonate born with an abdominal mass. On palpation, the mass occupied the right flank, and CT showed features suggestive of multicystic dysplastic kidney (MCDK). The patient had an ultrasound scan (USS)-guided right nephrostomy insertion for decompression, followed by a dimercaptosuccinic acid (DMSA) scan showing a poorly functional right kidney. The patient underwent nephrectomy, which showed a right MCDK along with proximal ureteral atresia. The patient was discharged on the sixth postoperative day and has been followed up in the outpatient clinic.

## Introduction

Ureteral atresia is a rare congenital abnormality of the urogenital system. It can be associated with ipsilateral renal agenesis or multicystic dysplastic kidney (MCDK) [1]. In 5% to 43% of MCDK cases, it may be associated with other genitourinary abnormalities; however, the incidence of MCDK and ureteral atresia is not specifically known [2]. In the case of our patient, CT initially showed features suggestive of a pelvi-ureteric junction (PUJ) obstruction as the cause of the right dilated, multicystic-appearing kidney. Intraoperatively, however, along with gross appearances of multiple renal cysts, a single, non-patent proximal ureter was discovered, with no evidence of a patent duplex system. This case report describes the rare occurrence of MCDK in conjunction with proximal ureteral atresia.

## Case presentation

A female neonate born at 37 weeks gestation via normal spontaneous vaginal delivery was referred to paediatric surgery for an abdominal mass. The mother, an asthmatic on beclomethasone, had a history of two stillbirth deliveries and one preterm delivery. She was of advanced maternal age and had an increased BMI. Three antenatal scans demonstrated gross right hydroureter and hydronephrosis; however, no reno-pelvic diameters were provided.

**Figure 1:**
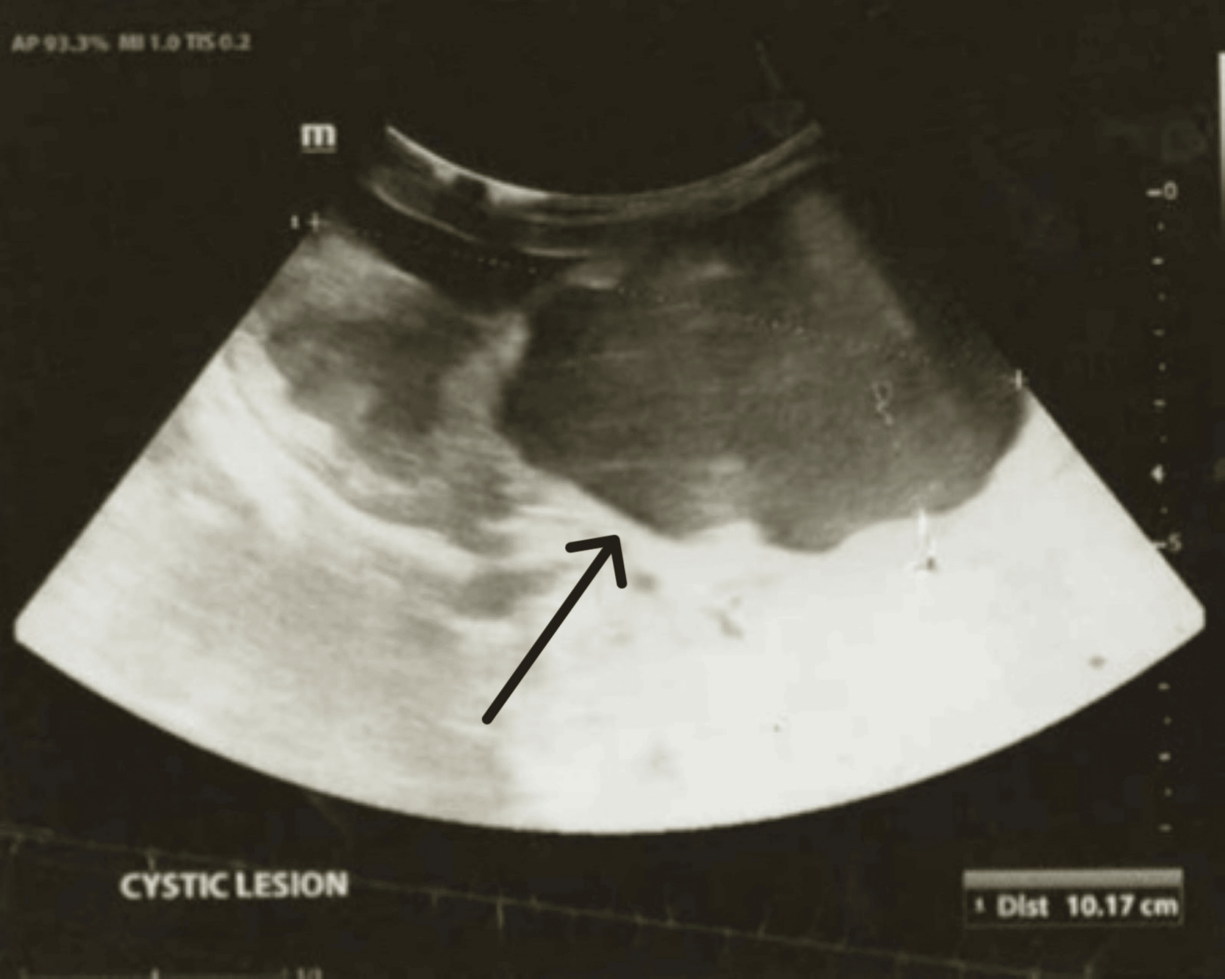
Ultrasound showing cystic lesion in region of right kidney (black arrow)

Upon delivery, the patient’s birth weight was 2.50 kg with normal appearance, pulse, grimace, activity, and respiration (APGAR) scores. Vitals were within normal ranges, and no obvious dysmorphic features were noted. No cardiac murmurs were heard, and the respiratory system was unremarkable. A firm right flank mass was palpated, not crossing the midline. The baby’s anus was patent, and a catheter was inserted into the urethra, which drained amber urine. The genitalia were in keeping with a normal female appearance. A nasogastric (NG) tube was inserted, and the patient was kept nil by mouth (NPO). Antibiotics were commenced, and abdominal X-rays and an ultrasound scan (USS) were ordered. The USS showed a cystic lesion with multiple septations seen on the right side of the abdomen, extending to the right hemidiaphragm (Figure 1). It displaced the small and large bowel to the left of the midline and the inferior vena cava (IVC) and aorta posteriorly. It measured 10.2 cm x 8.5 cm x 5.4 cm. The left kidney was visualized and appeared structurally normal, along with the other intra-abdominal structures.

A CT scan of the abdomen and pelvis was requested. The right kidney measured 9.4 cm x 7.6 cm. The left kidney was 4.4 cm x 2.7 cm. The right kidney showed gross hydronephrosis suggestive of PUJ obstruction with a multicystic appearance. All other organs appeared structurally normal, and no intra-abdominal free air or fluid was noted. Based on the CT result, drainage of the right kidney was planned. A trend of the patient’s blood results is shown in Table 1 below.

**Table 1:** Haematological values pre- and postoperatively

Blood investigation	Result (first admission)	Result on postoperative day two	Result on day of discharge	Reference ranges
Haemoglobin (g/dL)	17.1	12.6	14.6	9.5-13.5
WBC (10^3uL)	10.64	11.84	10.21	8-20
Platelet (10^3uL)	192	322	424	150-400
Creatinine (mg/dL)	0.8	0.4	0.4	0.5-1.0
Potassium (mmol/L)	4.5	5.1	5.0	3.5-5.1
Sodium (mmol/L)	140	130	141	135-145
Chloride (mmol/L)	105	98	111	97-110
Blood urea nitrogen (mg/dl)	6	5	2	35-104
Direct bilirubin (mg/dL)	0.5		0.1	0-0.40
Indirect bilirubin (mg/dL)	4.2		0.8	0-1
Total bilirubin (mg/dL)	4.7		0.9	0-1.20
C-reactive protein (mg/dl)	0.1	20.4	2.1	0-0.5

**Figure 2:**
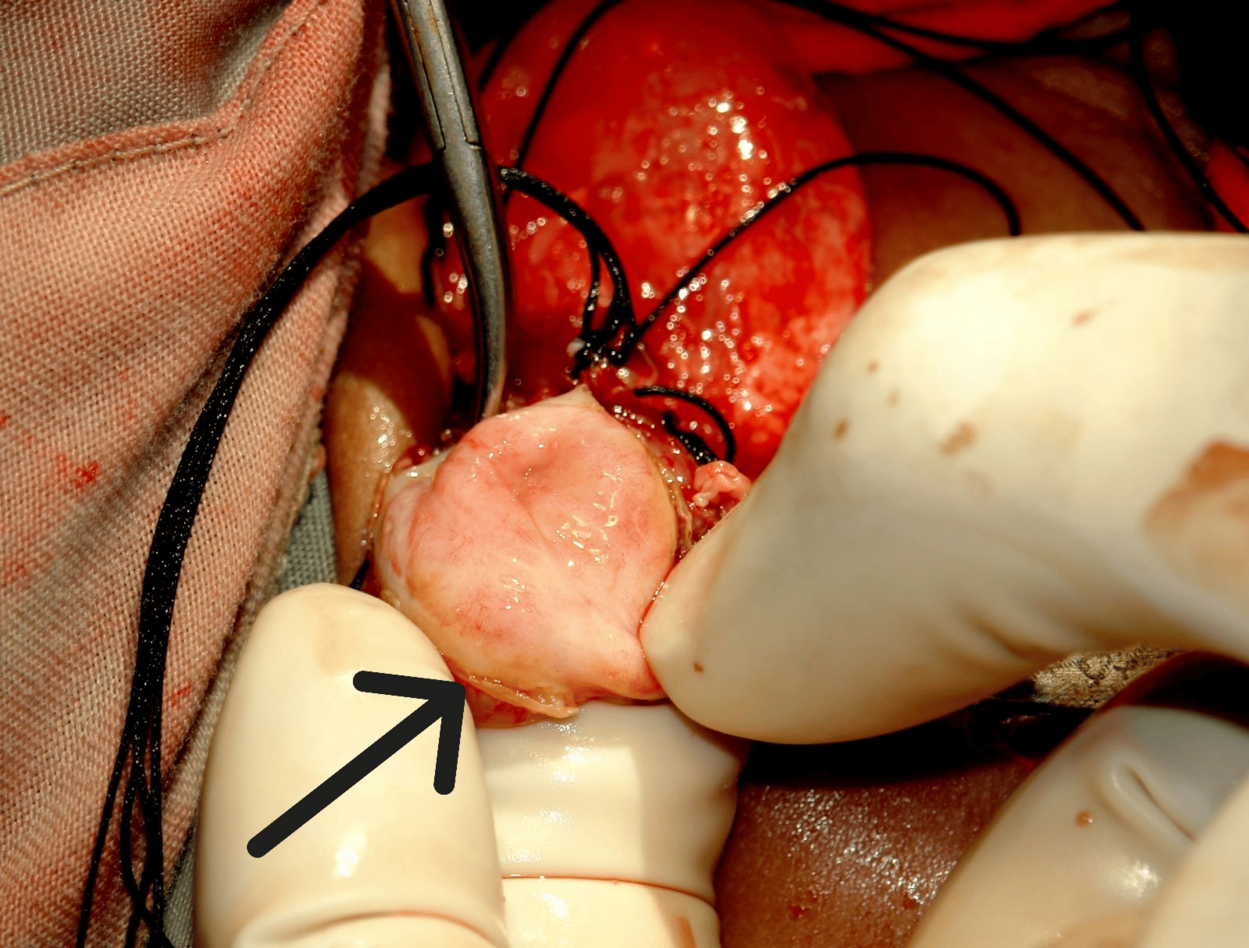
Intraoperative findings Black arrow: Proximal ureteral atresia noted at renal hilum

On day six of life, a nephrostomy tube was inserted into the right kidney under USS guidance, but poor decompression was observed. Urine cultures yielded no bacterial growth. After nephrostomy insertion, a dimercaptosuccinic acid (DMSA) scan was done, showing the right kidney function of 17% and left kidney function of 83%. Due to poor renal decompression, operative intervention with pyeloplasty was planned. A right oblique transverse flank incision was done, and entry was made into the retroperitoneum. A large, right multilocular dysplastic-appearing kidney was identified and decompressed, and perirenal adhesions were divided. A blind ending structure with no distal continuity was observed at the renal hilum, consistent with proximal ureteral atresia (Figure 2).

**Figure 3:**
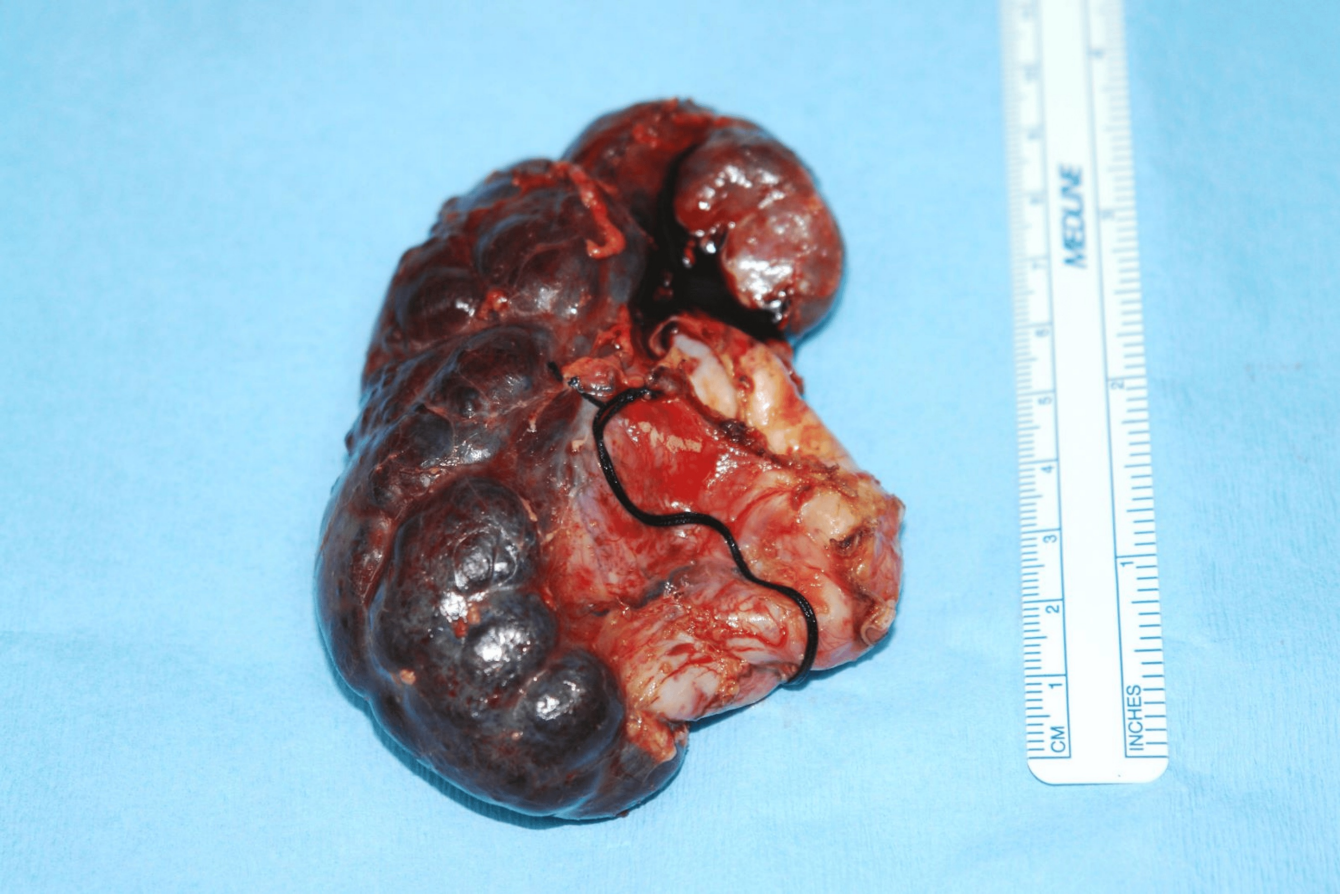
Fully excised, decompressed, right MCDK MCDK: Multicystic dysplastic kidney

Since the intraoperative finding of MCDK and proximal ureteral atresia was discovered to be different from the expected PUJ obstruction, the decision to do a right nephrectomy was made. The right kidney was mobilized and removed after the renal artery and vein were ligated at the renal hilum (Figure 3). No right ureter was seen entering the bladder. The cavity was irrigated, and an active drain was inserted, and the wound was closed in layers.

The patient was returned to the neonatal intensive care unit (NICU). She was placed on dopamine due to low mean arterial pressures (MAP) for age and left ventilated until the third postoperative day. By day five postnephrectomy, the patient had established breastmilk feeds, had normal renal function and stable haemoglobin levels, and was discharged to follow up in the paediatric surgery outpatient clinic (PSOPC). Histology showed MCDK with features of urinary obstruction, thus justifying the need for nephrectomy. The patient continued to thrive, with normal creatinine levels, and is growing along the normal parameters for age.

## Discussion

Ureteral atresia is a rare occurrence with poorly defined incidence. Garnaik et al., in 2025, reported only 15 cases documented worldwide thus far [3]. This condition may involve any part of the ureter. It may be bilateral or unilateral, focal short or long segment, distal or proximal, with distal ureteral atresia being the most common [1]. It results from the failure of development of the ureteric bud, and its association with other urinary abnormalities is rare. Embryologically, on day 28 of gestation, the ureter bud from the caudal end of the Wolffian duct moves into the metanephric mesoderm and shifts inferiorly until it connects to the future bladder. The ureter, renal pelvis, calyces, and collecting ducts are all derivatives of the ureteric bud, and alterations in development and migration give rise to conditions such as ectopic ureter, renal pelvis or ureter bifurcation, or complete urinary duplication [4].

Ureteral atresia may be due to an ischaemic insult during elongation of the ureteral bud or kidney migration, resulting in a failure of canalization of all or part of the ureter. Another theory is the failure of resorption of Chwalla’s membrane (a two-layered membrane transiently separating the ureteric bud from the bladder, which gets resorbed later during development) [5]. This occurs usually in conjunction with an ipsilateral dysplastic or absent kidney. Reflux may occur in the contralateral kidney, so voiding cystourethrogram (VCUG) is recommended as part of the initial evaluation [4]. Distal ureter atresia is more common; however, our patient’s atresia involved the entire ureter beyond the hilum, making this case even more unusual.

Ureteral atresia may manifest antenatally as fetal hydronephrosis. Postnatally, it may present as an abdominal mass due to renal obstruction [4]. As highlighted by Kaganstov et al., some patients may remain asymptomatic or have an atypical presentation later in life, with ureteral atresia being an unexpected diagnosis [6]. Preoperatively, however, ureteral atresia is difficult to diagnose [1]. If suspected, retrograde or anterograde pyelography is considered the gold standard imaging modality. Although many cases remain asymptomatic and undetected until adulthood, the dysplastic, obstructed kidney may predispose to infection.

Multicystic dysplastic kidney occurs in approximately one in 4300 live births. In the setting of ureteral atresia, it is thought to arise as a sequela of severe obstructive hydronephrosis. Another theory suggests that abnormal interaction between the metanephric blastema and ureteric bud leads to a failure of normal differentiation of these structures [7]. Recently, research has shown the benefits of conservative treatment of MCDK, as it is thought to undergo natural involution. Serial USS is recommended for monitoring size changes and to assess the contralateral kidney for evidence of hypertrophy [2]. The caveat, however, is that the course of MCDKs is unpredictable, and additionally, nephrectomy is more cost-effective than serial follow-ups as part of the conservative arm of management [1]. Given the poor socioeconomic status of our patient and the limited resource setting of our healthcare system, conservative management was not feasible. In our patient, an open nephrectomy was performed; however, laparoscopic nephrectomy has been proven to be safe, with favorable outcomes and better cosmesis compared to open nephrectomy [8].

Other associations with ureteral atresia and chronic obstruction include hypertension or tumors, therefore making a case for nephrectomy. In cases of renal preservation, pyeloplasty or uretero-uretero anastomosis have been successfully performed [1]. Ileal conduit or even ureteral substitution with the appendix are other options for renal preservation [9]. In this case, the indication for nephrectomy included the presence of a high proximal ureteral atresia with no tubule remnant that could be mobilized, canalized, or anastomosed in any way to achieve communication with the bladder, in conjunction with MCDK. Additionally, the DMSA showed a poorly functioning dysplastic kidney with an unsatisfactory response to diuretic administration, thereby justifying nephrectomy instead of a renal-sparing operation [1]. Furthermore, the patient’s unfavorable socioeconomic background did not allow for regular follow-up to assess for MCDK involution. Information on the long-term outcomes of children with ureteral atresia is limited, as this condition is rare and documentation is sparse. 

## Conclusions

Proximal ureteral atresia is exceedingly rare and therefore poorly documented among the paediatric population. This case highlights the presence of proximal ureteral atresia associated with MCDK. Surgical management involving nephrectomy served to eliminate multiple possible complications of MCDK. The involvement of a multidisciplinary team in the diagnosis and treatment of such a rare condition is imperative to providing the best quality of care to the patient. In our setting, nephrectomy was the best option based on the socioeconomic limitations of the patient in a resource-limited setting.
